# Foreign Summary

**Published:** 1839-04-27

**Authors:** 


### FOREIGN SUMMARY.

*On the Treatment of Syphilis.* By Professor Graves.—Notwithstanding all that has been done to illustrate the pathology and treatment of syphilis, it must be confessed that these subjects are still involved in much difficulty and doubt. A fact so incontestible, and so much to be regretted, makes it the imperative duty of every clinical lecturer to contribute whatever materials his experience may supply in elucidation of questions so important. For this reason, I have been induced to lay before you these observations on detached points of interest connected with the venereal disease. I shall, therefore, beg leave to direct your attention at present to the case of a woman, lately admitted into our wards, labouring under syphilitic iritis. From the history of her symptoms we learned, that, after a primary venereal affection, she got pains principally affecting the joints of the upper extremities, and aggravated at night. About a fortnight after admission, she was attacked with papular eruption and syphilitic iritis. I beg you will recollect the character and order of this woman's symptoms: at first, she would not admit the existence of a venereal taint, stating that her pains were only rheumatic, and that she knew no cause for them, except cold. Now, in her case, the arthritic affection was seated chiefly in the smaller joints; one of her wrists, and the hand and finger joints, were swollen, tender, and painful, and, at the first glance, had a very strong resemblance to the hand of a person labouring under rheumatic arthritis. It is generally believed that pains of a syphilitic character occupy chiefly the shafts and ends of the long bones; but in this instance we find that syphilitic inflammation may give rise to swelling, tenderness, and pain of the small joints, corresponding in many points with what has been regarded as rheumatic inflammation. We have another case of syphilitic inflammation of the synovial membranes and joints in a young woman in the small wards; but in this case, the larger joints are chiefly affected. It is absurd to suppose when a general disease like syphilis produces pains and inflammatory swellings, that they should be always limited to the long bones or their periosteum, for we find many instances in which the synovial membranes are also engaged. A point worthy of notice in this case is the manner in which the iritis appeared. We were treating the woman for the pains I have just alluded to, when she was attacked with iritis in a very insidious manner. There was scarcely any pain over the orbit, vision was but slightly impaired, there was no remarkable alteration in the state of the pupil; in fact, with the exception of some intolerance of light, and some conjunctival redness, there was scarcely any thing to indicate the occurrence of iritis. But whenever a person suspected to labour under syphilis gets inflammation, particularly if limited to one eye, no matter whether it commences in the internal or external tissues, you should watch it closely, for the chances are, that it will prove syphilitic ophthalmia, endangering vision. And such was the result in this case; for in four or five days the woman exhibited symptoms of decided iritis. It has been very properly remarked, that the name syphilitic iritis is calculated to mislead; for the iris, in many cases, is not the part principally or primarily attacked; and, in some instances, it appears to escape entirely, although the vision is lost. Syphilitic ophthalmia appears a better name for this affection.

There is scarcely any disease which occasionally proves so insidious in its approach as syphilitic iritis, nor is there any form of internal inflammation more variable in its progress, degree, or intensity. Sometimes it commences internally, attacking in the first instance the tissues of the iris and the adjoining parts, proceeding in its course with remarkable intensity, and destroying vision completely, if not arrested at once. In such cases it is accompanied by severe pain, intolerance of light, lacrymation, and increased vascularity of the sclerotic, so that no one can mistake it; but, at other times, its approach is so insidious, and its progress so slow and painless, that vision of one eye is lost before the patient is aware of it. The iris is then seldom engaged until a late period of the disease; and the slow inflammation, by which vision is ultimately destroyed, commences in the deep-seated tissues of the eye. In many cases, as in that now before us, it takes a contrary direction, commencing in the external parts of the organ, and being usually ushered in by conjunctivitis, apparently simple, and produced by cold. Hence, you perceive, there is a great variety as to the mode of origin, progress, and intensity of syphilitic ophthalmia, and from this you will infer that there must be some diversity in the treatment. The physician is to be chiefly guided by the intensity with which it attacks the eye, and hence the treatment which would be proper for one case would be wholly unfit for another. I am anxious to advert to this matter, as I think we did not treat the case of this woman as we ought to have done, had we considered its nature more attentively. If syphilitic ophthalmia be of an intense character, attacking the iris and lens at once, and threatening to destroy vision in a few days, the activity of our treatment must be proportionate to the imminence of the danger; we must bleed, leech, and give calomel and opium in large doses, say ten grains twice or three times a day, and must continue its administration until the mouth is affected. In this instance, a disease that would destroy vision in three or four days, is cured in the same space of time, and the activity of our treatment is adapted to meet the intense and rapid character of the ophthalmia. We produce full salivation in as short a time as possible, and apply the extract of belladonna to the eyelids, to keep the pupil from contracting. In syphilitic iritis there are many shades of intensity, and the treatment must correspond with the existing symptoms. Now, if the disease be of a chronic nature, and has advanced slowly, it must be made to recede slowly. You should endeavour to remove it by the gradual ingestion of mercury, aided by the usual local means. In the former case you have only three or four days for action; in the latter you have as many weeks. Hence, I think, we were too precipitate in our treatment of this woman. Her disease came on slowly, and without violent or urgent symptoms, consequently we ought to have treated her mildly, giving small doses of calomel or blue pill, so as to bring the system gradually under the influence of mercury. But we salivated her at once, and the consequence was, that although she improved at first, the disease became afterwards exacerbated. Had salivation been gradually superinduced, the relief obtained would have been less speedy, but more certain and permanent.

You will, therefore, whether you treat syphilitic iritis, or syphilitic pains and periostitis, or sore throat, or eruption, be guided by the character and progress of the symptoms. If the disease has come on gradually—if it be mild or chronic in its nature, and no vital part threatened—you may take time, and proceed gradually in mercurializing your patient. But where the vitality of any organ or part is endangered, you must act with promptitude, and throw in mercury, as it is termed, at once. Thus, where syphilitic ophthalmia attacks the eye in such a manner as to be likely to destroy vision in a few days, it will be necessary for you to give five or ten grain doses of calomel, three times a day; and the same line of practice will be required when periostitis attacks the orbit, particularly the thin plate of bone between the eye and the brain, or when it fixes itself in the internal table of the cranium, and threatens the dura mater.

I may observe here that a consideration of the nature of those tissues, in which scrofula is most commonly developed, will give you much information with respect to the administration of mercury in venereal affections, and the energy with which this agent is to be employed on various occasions. The vitality of the white tissues is low, and their inflammatory affections of a more subacute and chronic character; and hence not demanding such energetic treatment as where tissues of a higher order are attacked. This you may lay down as a general rule. But there are some exceptions, as in the case of an organ composed of various tissues, as the eye; or when it attacks purely albuminous tissues in a very acute and intense form. In general, the vitality of periosteum and bone is low, and so is that of most of the tissues of the eye; and whenever you have to treat inflammations of such parts, you should not expect to be able to produce any sudden change, for parts of this description require a considerable time for the restoration of their healthy functions. Hence, in the majority of cases, periostitis and syphilitic ophthalmia, with the exceptions already alluded to, are to be removed by a mild alterative treatment, by small doses of mercury and gentle frictions, so that some weeks shall elapse before the mouth is affected. Nor should you attempt to bring on full salivation: touch the gums slightly, and keep them in that state for some time, exhibiting as much mercury as will just keep its influence in the system.

I have already devoted some lectures to the consideration of periostitis, and it is unnecessary to refer to it again; but I may observe, that you will require considerable discrimination to determine in some cases whether the affection you are about to treat is syphilitic or not. You will find many examples of periostitic inflammation depending wholly on a scrofulous taint in the constitution; for scrofulous inflammation is often fugitive, and attacks the periosteum before it fixes in the bones. You may also have periostitis from rheumatism, or from gout; but one of the most common causes of periostitis, in persons not labouring under syphilis, is connected with the secondary effects of mercury on the constitution. Persons who have taken mercury for any disease, no matter whether it be pneumonia, pleuritis, or hepatitis, are afterwards subject to periostitic inflammation, and this liability continues not for months, but even years. Indeed, periostitis is one of the most common effects of mercurialization, particularly if the patient be exposed to cold while taking mercury. In the course of one, two, three, five, or even a greater number of years, exposure to cold, a blow, and other apparently trivial causes, will give rise to periostitis in some individuals. I am at present attending, with Mr. Crampton and Mr. Cusack, a gentleman labouring under periostitis of the tibia and cranium; and on inquiring into the history of his case, we find that it is nearly nine years since he was salivated. I have also witnessed a very severe case of periostitis affecting the shafts of both tibiæ in a lady who took mercury about five or six years ago for supposed hepatitis. One of the most remarkable cases of periostitis after mercury which have ever come under my notice, I have recently witnessed in the person of a gentleman who was for some years surgeon to the British Envoy to Mexico. In that country, raised nearly twelve thousand feet above the level of the sea, and exposed at once to sharp winds and a burning tropical sun, fevers of an intense character often prevail. Some time after his arrival, this gentleman was attacked with fever, for which he was fully salivated. He caught cold during his convalescence, and was attacked with periostitis, for which he took mercury again with relief. Next year he caught cold again, was again attacked with periostitis, and cured by mercury, as before. The year after, the same series of accidents was repeated. I forget how many successive attacks he had, each originating from cold, and each, like the former, removed by mercury. At length the mercury seemed to lose its power over the disease, and was no longer capable of relieving it. He returned to this country with the view of improving his health by change of air, and presented a most extraordinary spectacle. The periostitis had chiefly fixed itself in the cranium, which it had altered so as to have no longer any resemblance to the human skull. When I saw him, a considerable portion of the pericranium and bones of the head had been affected with periostitis for three years, without any intermission. His skull would have defied the scrutiny of Gall or Spurzheim, for its shape was the most extraordinary I ever witnessed. He was in the habit of taking large quantities of opium to procure some alleviation of his sufferings, and was restless to such a degree that he was frequently for fifteen or twenty nights together without an hour's sleep. Altogether he was in the most pitiable state; and seldom got any relief until the attacks were wearing off, when he enjoyed some brief intervals of repose. Some fifteen or twenty years ago, when the subject of the treatment of syphilis was warmly canvassed, it was asserted by the mercurialists that mercury never gave rise to nodes or periostitis, unless where there existed a syphilitic taint in the constitution. Now I can attest from manifold experience that this is not true. The gentleman whose case I have related had never been affected with syphilis. But there is no necessity of insisting on this point. Every practical physician knows that mercury may and does give rise to a train of symptoms bearing some analagy to those of secondary syphilis. Thus, after the use of mercury, a patient may be attacked with feverishness, pains in the bones, nodes, sore throat, and an eruption, to which the name mercurial eczema has been given. Here, you perceive, we have a remarkable analogy between the diseases produced by mercury and syphilis. Mercury, when exhibited improperly, may produce all the affections I have enumerated, and in addition to these caries of the bones, particularly of the nose and palate. It is well known that some active remedies have a tendency to produce diseases somewhat analogous to those they are known to cure. This is frequently observed with respect to mercury, belladonna, strychnia, quinia, hydriodate of potass, and some other powerful medicinal agents. In fact, it is hard to expect that a remedy will cure a disease affecting a certain tissue or tissues, unless it has some specific effect on such tissues; and in this point of view we have an example of the "*similia similibus curantur*" of the homœopathics.

Mercurial ostitis of the head is a very common form of disease: its more usual seats are the frontal and parietal bone; but it is sometimes observed also on the other bones of the skull. In general, the inflammation affects the external table of the bone, and is then easily recognised from the tenderness and swelling of the corresponding portions of the scalp. Sometimes, however, the inflammation commences in the internal table of the skull, and where this occurs the disease wears a much more alarming aspect, for it is then apt to implicate the dura mater and subjacent portion of the brain. In such cases, the true nature of the complaint is not unfrequently overlooked, or mistaken for some other disease causing headache. This is a very serious and fatal error; for unless the physician is aware of the real nature of the malady he has here to contend with, he will seldom adopt proper measures, and the patient will fall a sacrifice. Such cases are indeed obscure, but we may in general make out their true nature by a careful attention to their history. Thus, if severe nocturnal headaches arise in a person who has ostitis in other bones, and if the pain darts from some fixed point, then, although all external tenderness be wanting, we may safely conclude that the cerebral affection originates in ostitis of the cranium. In investigating such cases, I have derived much advantage from percussion. I place the back of one finger on the patient's head, and tap it smartly with the fingers of the other hand. If internal ostitis be present, every tap excites a peculiar internal pain in the part affected, which pain is the greater the nearer the part percussed is to the seat of the disease.

You have seen in our wards several men complaining of very agonizing headache without any external tenderness; and you have witnessed in these cases the failure of the common means for relieving pain in the head, and the success which followed the adoption of a treatment founded on a true diagnosis of the disease. This headache, yielding to no other species in severity, deprives the patient altogether of rest—occasionally occupying chiefly one side of the head—and most severe at certain hours, is not unfrequently mistaken for nervous hemicrania, and treated with iron! When ostitis occupies the external table of the cranium, it seldom strikes inwards, so as to engage the internal, and disorder the brain. That it does so sometimes appears from several cases; among the rest, that of Mary Wilkinson, admitted into our ward on the 21st of October. In her the scalp was excessively tender, and felt in one part thickened and boggy. There was dilatation and increased pulsation of the external arteries supplying that side of the scalp. On the 27th, the headache increased, and she fell into a state of profound coma, with dilated pupils, insensible to the light; the extremities were cold, and pulse scarcely perceptible. Luckily, while in this state, the mercury previously administered began next day to affect her mouth, and, aided by large doses of calomel, and powerful blistering, soon restored her. Such a recovery very seldom takes place. Ostitis is also very dangerous when it occupies the orbital and contiguous portions of the frontal bone. It is very obscure when seated at the base of the skull.

Mercurial ostitis is a very common occurrence in the cervical vertebræ, but comparatively rare in the dorsal. In the lumbar it becomes again more frequent, but not so much so as in the cervical. I have, however, seen some cases where the dorsal vertebræ appeared to be almost all engaged in the disease, and where, consequently, the greatest agony was experienced on their being touched or moved. Pathologists have not yet paid sufficient attention to the species of neuralgia which is occasioned by inflammation of the nerves or their sheaths, spreading from the surface of the bones through which they pass.

Nothing is more certain than the fact, that in many, the abuse or even the use of mercury renders the constitution disposed to ostitis on future occasions, when cold and damp act on the body, especially if fatigued by exercise, or exhausted by dissipation. This ostitis is consequently called mercurial: but this name must not mislead us; for, strange as it may appear, the disease often yields readily to mercury—a mode of treatment generally effectual for the moment, but attended with the obvious disadvantage, that it leaves the patient more liable than ever to future and severer relapses, which will at last refuse to yield to mercury.—*London Medical Gazette.*

---

*Strangulated Femoral Hernia—Operation—Cysts in the Fat under the Fascia Propria.* By Mr. S. Cooper.—Mary Eggleston, æt. 66, admitted December 19th, 1838. She is a washerwoman, who has had several children, and has been the subject of a femoral hernia of the right side for several years. She attributes its first occurrence to exertions made in mangling. Formerly she wore a truss, but discontinued its use about four months ago, since which period, until the day but one before her admission into the hospital, the tumour never reappeared. On the morning of the 17th, however, in consequence of her having made considerable exertions, the hernia descended again.

On the 18th, as the tumour was painful, she consulted Mr. Dore, a surgeon in my neighbourhood, who had recourse to the taxis, and administered some castor oil and other medicines. The taxis did not succeed; but one evacuation from the rectum was obtained. In the course of the night the pain in the tumour became more severe, and vomiting came on. On the morning of the 19th, the patient had a scanty motion, which afforded her no relief. The vomiting now became more frequent and distressing; nothing could be retained in the stomach, and hiccough was added to the other symptoms.

Mr. Dore called upon me on the evening of of the 19th, and asked me to receive her into the hospital, to which request I immediately acceded.

*Symptoms on Admission.*—The countenance was pale and anxious; the stomach retained nothing that was put into it; and there was a frequent and annoying hiccough. In the right groin, a tumour of about the size of a pigeon's egg, was detected below Poupart's ligament. It was tense, painful when handled, and lay with its greatest diameter in the direction of the fold of the groin. I pressed upon the abdomen, but it gave no pain—a very favourable circumstance, as denoting that peritonitis had not yet made any dangerous progress. The pulse was 90.

My house surgeon, Mr. Carter, tried the taxis in vain; and an ultimate trial of it was made both by Mr. Quain and myself, after the patient had been made faint by being put into a warm bath of 98°.

*Operation.*—I performed the operation at 9 o'clock in the evening, about two hours after the patient's arrival at the hospital. The first incision was begun at Poupart's ligament, and carried downwards and outwards directly over the centre of the tumour. On opening what appeared to me to be the fascia propria, I observed that two or three drachms of clear serous fluid escaped—an occurrence which, I believe, is not noticed by the best works on hernia, though Sir Astley Cooper informs me that he has met with it in practice. Then, on laying open the supposed fascia propria, a large mass of granular fat presented itself, which had much of the appearance of hypertrophied omentum. The finding of a mass of adipose substance in this situation, being rather common in fat subjects, would have occasioned no perplexity; but another circumstance, which was entirely new to me, and so far as my inquiries extend, has not been described in any treatise on hernia, was the presence of cysts in this adipose substance, a portion of one of which, projecting beyond the surface of the granular fat, and having a darkish appearance, looked very much like a piece of bowel enveloped in protruded omentum, and intimately adherent to it. I first tried to separate the adhesions with a scalpel, but found this quite impracticable with any degree of safety to the intestine, if it proved to be such. The texture of the protruded portion of cyst was then closely examined, and a very slight appearance of transparency was perceived in it when the candle was held on one side of it. This circumstance was remarked by Mr. Carter, and it convinced me that the part could not be intestine. I determined, therefore, with the concurrence of my friend, Mr. Quain, to puncture it; and thus about a drachm of fluid, which was not perfectly clear, was discharged. The cavity from which it came was next examined with a probe, and found to be completely circumscribed.

I then dissected carefully more and more deeply through the mass of adipose substance, and arrived at another similar cyst of a darkish colour, which was also opened, and at length the true peritoneal hernial sac exposed. As soon as this had been opened, a small quantity of turbid fluid gushed out, and a piece of intestine, of about the size of a walnut, and of a chocolate colour, presented itself. The stricture was now divided upwards and inwards, and the intestine reduced.

The wound was then dressed, and a compress and spica bandage applied. An injection was thrown up the rectum, and an effervescing draught given, with a few drops of tinct. opii. At 12 o'clock, P. M., the patient fell asleep, and did not awake till 2, when she became very sick, and had a motion.

20th.—Pulse 90; sickness diminished; no pain nor tenderness about the abdomen. At 5, P. M., as her pulse had risen to 100, twelve ounces of blood were taken away, which proved to be buffy and cupped.

Suffice it to add, that all functional disturbance soon ceased, and that, with the aid of one more bleeding, and medicines for the relief of a severe cough, the patient recovered, without having had, subsequently to the operation, any very dangerous symptom.

The peculiarity of this case excited, at the time of the operation, a suspicion that there might be two hernial sacs; but the account which I have given, seems to me the correct one; and it must appear to you, gentlemen, as it certainly does to me, that the presence of cysts in the adipose substance, which lay between the fascia propria and the hernial sac, constitutes the most interesting feature of this example of femoral hernia. I do not find such an occurrence noticed by the best writers on the subject; and as it may take place again, and cause perplexity, I deem this explanation of it to you, and even the public record of it in some form or another, a matter of duty.

Sir Astley Cooper acquaints me, that he has never seen any instance of cysts in the fatty substance within the fascia propria; nor has he ever met with any case of protrusion of two hernial sacs through the crural ring.

The only case which I know of, as resembling that which I have now related, was met with by Mr. Morton, in the dissecting rooms of University College, singularly enough, a few days after the foregoing operation. The following is an extract from notes which this intelligent surgeon made of what was observed :—

"While dissecting the inguinal region in the body of an old female, the subject of crural hernia on both sides, we found a small cyst, of the size of a walnut, lying in front of, and upon the peritoneal sac of the hernia of the right side. The membrane, forming this cyst, was very thin and delicate, resembling much of the appearance which is presented by some of the bursæ mucosæ. In the cavity of the cyst was found a small quantity of thin and clear serous fluid. The cyst was situated between the fascia propria and the peritoneal sac of the hernia, apparently in the sub-serous cellular tissue.

"Dr. Sharpey and Mr Quain saw and examined the cyst soon after it was exposed."

*Lon. Med. Gaz.*

---

*Medical Jurisprudence.*—The determination of the period since which a fire-arm may have been discharged, is a point of much importance in medical jurisprudence, and evidently applicable to various cases of homicide, wounds, &c. The question has recently been examined with much care, by M. Boutigny, who has ascertained, by numerous experiments, that we can indicate very closely the period at which a fire-arm has been discharged. It may, however, be objected that as the barrel of a gun may be easily washed, all traces by which the medical jurist is guided may thus be obliterated. M. Boutigny has provided against this objection, or rather determined the characters by which it may be known whether a gun-barrel has been recently washed or not. The author has discovered that the iron of a gun-barrel does not become oxidized for a considerable time, whenever the interior of the barrel has been lined, as it were, with the residue of the combustion of powder; and even when oxidization does take place, the traces escape the naked eye, because the oxide is gradually dissolved in the acid of the sulphate of potash, or in that resulting from the oxidation of the sulphuret of potassium. Hence it follows that the wadding of the gun will present certain differences, according as the gun may or may not have been washed before having been charged.

We must refer the reader to the original article, ("Ann. d'Hyg. et de Med. Legal.," January, 1839,) for an account of the experiments of the author, whose conclusions only we here insert.

The wadding of a gun which has been reloaded without having been washed, presents a grayish-black tinge; but if the gun have been cleaned, the wadding is of an ochre or deep reddish colour. However, when a gun has been charged immediately after having been washed, and the wadding is examined a few hours afterwards, the colour is then found to be a greenish-yellow, which passes rapidly to a brown-red, when exposed to action of the air and atmospheric moisture. If to the preceding characters we add those which are derived from the absence or presence of sulphuric acid, we may conclude to a certainty that the gun has been cleaned or not, before it has been charged. In order to render the materials which are to be submitted to the medical jurist available, certain precautions must be taken by the magistrates or police authorities into whose hands the suspected arms may, in the first instance, fall. The muzzle of the gun should be closed with a paper wadding, and then covered over with some paper, to which an official seal should be attached. The same precaution should be employed with respect to the lock of the gun, whether it be a flint or percussion one. *Lancet, from Arch. Gen. de Med.*, Feb. 1839.

---

*Observations on the Nature and Treatment of Nævus.* By Frederick Tyrrel, Surgeon to St. Thomas's Hospital.—The author begins by observing, as the result of much experience in the treatment of this disease in the last few years, that of the many plans of treatment which have been suggested by their inventors, none are exclusively applicable to every form of the disease. His object, therefore, in his present communication, is rather to point out the description of a case to which each method is adapted, and to indicate the rationale of its action, than to offer any new plan of his own. With this view he considers—1st. The nature of the disease. 2d. Its varieties, pointing out the seat, the position, the progress and consequence of each form, if allowed to run its course. 3d. The different modes of treatment in present use; and 4th, the proper application of these means. When the disease is purely cutaneous, not extending at all to the subjacent cellular texture, he recommends the forming a belt around its margin on the sound skin by means of concentrated nitric acid, and afterwards imbuing the surface of the growth with the same liquid: at once, if small; but if of great extent, by repeated applications made to a small portion at a time. The author holds, however, that the use of escharotic applications should be confined to those cases which are purely cutaneous, since in those which extend more deeply, the agency of the acid stops short of the deeper seated parts of the tumour, and consequently, when the superficial part separates by the ulcerative process, hæmorrhage may be expected to ensue. For the destruction of the subcutaneous form, as well as that of a mixed character, he recommends the injection into their substance of stimulating fluids; but he points out a very important preliminary step, which, in his opinion, will prevent those accidents that have sometimes attended the too wide diffusion of the injected fluid; viz., suppuration and unsightly puckering of the skin after the cure. This plan consists in cautiously injecting a small portion of a saturated solution of alum into the surrounding cellular tissue, before anything is done to the nævus itself, with the view of producing its consolidation, and thus preventing the extension of the disease by the excitement to be afterwards induced in the tumour by the injection of the stimulating liquid into its own substance, as well as the undue diffusion of the fluid. Cases are detailed of the successful employment of this practice. The author speaks highly of the ligature, as a means of relieving a great variety of forms of nævus, but expresses his fear that setons passed through the substance of the tumours may be productive of hæmorrhage, which, in young and delicate subjects would be dangerous.

Sir B. Brodie had treated small subcutaneous nævi, in situations where it was advisable to avoid the scars which would follow the use of the ligature or the knife, in the following way:—He melted some nitrate of silver in a platinum spoon, and dipped into it the blunt points of two or three probes, which, being withdrawn in the space of a few seconds, were found to be coated with the caustic; he then made one, two, or three punctures, according to circumstances, in the nævus, by means of a small instrument resembling a lancet, and into these punctures he inserted the armed probes, and allowed them to remain for a minute or two, until the nitrate became decomposed by acting on the structure of the nævus; he had a little oil in readiness, in order to counteract the too violent effect of the caustic. In this way inflammation was set up, and the tumour became consolidated. In general, one operation was sufficient to effect a cure, in other instances, the proceeding required to be repeated twice, or more frequently, the pain attending which was very slight. In the case of a child, who had a large nævus extending over the greater part of the face, and in which a variety of means had been resorted to, the application of nitric acid among the rest, he had pursued the above plan in a part of the tumour; in the other portion he had broken up the net work of vessels, by adopting the proceeding recommended by Dr. M. Hall. A perfect cure ensued, although an ugly scar remained on the part to which the nitric acid had been applied. He had also treated, successfully, by this mode, a case of an ugly sub-cutaneous cellular nævus situated at the extremity of the nose. He punctured it in several parts, and then introduced the probes. Some slight puckering of the skin where the caustic had been inserted, were the only marks which remained. Whilst speaking on this subject, he might also allude to another kind of marks very commonly found upon the face, and consisting of little stellated patches of blood-vessels. Generally speaking, these went away when left alone, but persons in high life frequently complained of them as blemishes, and requested means to be adopted for their removal. When looking at these spots through a glass it was easy to discover one or two larger vessels entering into, and supplying the net work, which spread out like the web of a spider. Having found the supplying vessel or vessels, he placed on them the end of a small probe, and if he found that the red spot entirely disappeared, he proceeded thus: he divided the vessel by a minute puncture, and then destroyed it by inserting a piece of caustic potash scraped to a very fine point; he then introduced a small quantity of vinegar, in order to prevent the caustic extending its influence beyond a certain limit.

Mr. Cæsar Hawkins, in allusion to the use of steel needles, as recommended by the author of the paper, in the treatment of nævi by ligature, suggested that the old silver needles would not require the nipping off of their ends, and would not, therefore, be so likely to produce irritation. Nævi generally consisted of a mixture of arteries and veins; occasionally, however, they were entirely venous. He had seen a congenital case of this kind, in which the disease occupied the back of the head and neck, and extended down as low as the scapulæ,—the tumour consisted of branches given off from the post-aural, occipital, and lingual veins. There was no discolouration of the capillaries, and no pulsation. The child was now seven years of age, and the tumour increasing.

Mr. T. B. Curling rose to notice one of the objections which the author of the paper had advanced against the treatment of nævi by setons, and which consisted in the fear he entertained of the occurrence of dangerous hæmorrhage. Now he (Mr. C.) had used the seton in a great number of cases of nævi, no hæmorrhage, except such as was readily stopped, taking place; he thought indeed, the great advantage of the treatment by setons, consisted in its freedom from the occurrence of hæmorrhage. The treatment by ligature was objectionable; for, even though it might cure, it left an ugly scar, and was not free from danger. In a case in which the crossed ligatures were applied, the child perished in a few days, from the occurrence of great constitutional irritation. The mode of treatment by injection, he believed, was originally proposed by Mr. Lloyd. There was one source of danger from this proceeding, for as it was necessary that very strong caustic should be employed, there was fear of its making such an impression on the larger vessels as to be attended with danger. In a case treated on this plan, the patient died almost instantly, probably from the above cause. In what way did Mr. Tyrrell guard against the occurrence of such an accident? Mr. Lloyd, for this purpose, had recommended the use of a piece of pasteboard, with a piece the size of the disease removed from it.

Mr. Tyrrel considered that the explanation given in the paper, of the precautions he took for preventing the occurrence alluded to by Mr. Curling, was sufficient. He (Mr. T.) had there stated that he invariably consolidated the surrounding cellular tissue before he interfered with the tumour itself. In two cases in which he had employed the injection, the disease was much reduced in size before he touched it, proving the influence of the consolidation around. He thought this plan quite as successful as the one recommended by Mr. Lloyd. He (Mr. T.) did not bring forward his plans as perfect, but merely as the result of his own experience. He regretted that in the reading of the paper the secretary had left out the most important case, which had been treated by the application of nitric acid. In this instance the nævus was cutaneous, occupying part of the superior eye-lid, the whole of the left cheek, half of the upper lip, and extended up to the septum nasi. Tartar emetic had been employed, and part of the disease had been destroyed by it, but it was extending in other directions. He circumscribed the boundary line in this case, the nævus being very large, at two distinct periods. This proceeding was followed by no extension of the disease. He then painted over the surface, piecemeal, with a brush dipped in the acid, and touched the neighbouring portion to the last, at each successive application. In this way hæmorrhage was avoided, and, after five or six applications, the disease was removed, there being only, here and there, a slight contraction of the skin, consequent upon the use of the tartar emetic.—*Lancet.*

---

*On the Endermic Application of the Salts of Morphia.* By Prof. A. T. Thomson.—In lecturing on these cases, Dr. Thomson said that the pupils had had many opportunities of observing the plan which he had pursued in these cases,—and which he had also extensively employed in private practice. He was anxious to offer a few remarks upon the subject, and to explain the manner in which he conceived narcotics operated, when they were thus administered. If we reflect (said he) how long a period has elapsed since medicinal agents had been applied to, or rubbed upon, the entire skin, and how much benefit had been derived from counter-irritants, it was remarkable that no advantage had been taken of the skin, as an inlet to medicinal subtances capable of acting generally upon the system, until the year 1823. M.M. Lembert and Lessieur, at that time suggested what was termed the *endermic method.* They had some followers among continental practitioners, but few in this country had ventured to administer medicines in this way; and it was not too broad an assertion to affirm that it had been most unjustifiably neglected. Since he, Dr. T., had had the honour of being one of the physicians of the University College Hospital, he had lost no opportunity of essaying the value of the endermic method, and he had seen such advantages derived from it, that he was most anxious to recommend it to the members of the profession, as well as to the pupils of the hospital.

He was fully aware that, in clinical instruction, hypotheses should be avoided; but, at the same time, the explanation of the manner in which remedial agents influenced the habit, if founded upon correct physiological principles, could not fail to be acceptable. His theory of the *modus operandi* of narcotics, when applied to the skin, previously deprived of its epidermis, was based upon that foundation; he did not claim for it more than its legitimate value. But, before explaining his opinions, he would mention a few circumstances relative to the best method of denuding the surface, and preparing the skin to aid the influence of the narcotics which might be applied to it.

The most common method of raising the cuticle was the application of a blister; and since the introduction of the acetum cantharidis, we possessed a very rapid and efficient means of blistering. This might be also rapidly effected by means of a compound of four parts of lard rubbed up with six of *strong* liquid ammonia. The best mode of removing the cuticle was to apply over the blister an emollient poultice; the whole of the raised cuticle was, by this means, taken off, without that suffering, to nervous and irritable patients, which the ordinary method induced. The salts of morphia, when these were the narcotics employed, tended, in some degree, to promote the suppurative process on the denuded surface, and, consequently, to prevent it from cicatrizing. Opium, henbane, and belladonna, operated in the same manner; but he had found that the influence of all of them, in that respect, was greatly augmented by the addition of a small quantity of refined sugar. It was not necessary to apply a large blister; on the contrary, as the quantity of a narcotic to be applied was small, the denuded surface needed not to be greater than it could cover.

The full dose of the narcotic should not be applied at first, the irritant influence of some narcotics being so great as to cause inflammation in the part, and thence to check absorption. It should be gradually augmented as the habit got accustomed to it; and when the desired effect had been produced, the dose should be as gradually diminished. With respect to the part of the skin to which the narcotic was to be applied: when the local influence only was required, the blistered surface should be that directly over the seat of the pain; when the general effect was to be produced, it should be as near to the head as possible. One advantage of the endermic method over the internal administration of powerful narcotics was, that of obtaining the full influence of the narcotic without any chemical change being produced upon it, such as must, necessarily, often occur when it was taken into the stomach: another was, that the digestive function was left undisturbed: and a third was, the power which we possessed of restraining the too great activity of the narcotic by the application of an exhausted cupping-glass over the part to which it had been applied. For, although this would produce no advantage if the whole of the narcotic was already taken into the system, yet if the hurtful influence of it was displayed when it had been only partially absorbed, the cupping-glass effectually checked its farther absorption.

Besides narcotics, he had advantageously administered other remedies by the endermic method; namely, strychnia, extract of colchicum, and iodide of iron; but he should confine his present remarks to the salts of morphia. In the greatest number of cases the hydrochlorate was employed; but, when an anodyne influence was especially desired, the acetate operated better, more efficiently in allaying pain, and in a shorter period of time than the hydrochlorate, probably owing to its deliquescent property. When the salts of morphia are applied to a denuded surface, they excite a burning heat in the part, a fact which might, *à priori*, be expected, as all stimuli cause sensation in nerves, whether entire or mutilated, so long as the irritated portions maintain their connection with the brain or the spinal chord. In this case, the chemical irritant property of the salt acts on the excitability of the nerves, and causes pain; but, after a short time, the effect thus produced ceases, and the sensibility of the nerves is deadened. Some change, although not an obvious one, is produced in the material composition of the nerves; a loss of susceptibility of impression is the consequence, and neuralgic pains are thus lessened, or they wholly disappear. Now, were narcotics administered internally in doses sufficient to produce such effects, they would give rise to the same injurious consequences as result from morbid or excessive stimuli, and they would tend to annihilate the vital force; whereas, when endermically used, much of their influence is exhausted in their local action on the affected nerves. That such a local action exists there could be no doubt; for, independent of the experiments of Humboldt, Müller, Wilson, Phillip, Brodie, and others, which had demonstrated the local influence of narcotics on the nerves, we cannot ascribe to any general action, nor to absorption, either the influence of belladonna upon the iris, when it is applied to the eyelids, or that of carbonate of lead upon the wrists of those who work in lead. In these cases, although the narcotic reaches the nerves of the iris and those of the wrists by imbibition, yet it is evidently a local influence which is exerted, for the pupil of the other eye remains unaffected, and although the wrists are paralyzed, the power of volition over the nerves continues. Were any other proof requisite, he had only to mention the experiment of Müller. He dissected out the ischiatic nerve in toads, and left the leg connected to the body only by that nerve. He next immersed the leg and nerve in a strong watery solution of opium; both were paralyzed; no contractions of the muscles could be excited by the galvanic, nor by any chemical stimulus. In another experiment, he immersed the bared nerve only in the solution of opium, after which the galvanic or a mechanical stimulus excited no contractions in the muscles, when applied to the upper part of the nerve, but convulsions were produced when the nerves were excited from below,—a fact clearly demonstrative of the local influence of the narcotic. It is still further probable that in every instance where there is a general action of the narcotic, this is dependent upon its absorption; or, in other words, that it operates through the medium of the blood, whether the narcotic be taken into the stomach, or applied to a denuded surface. It might be supposed, however, that, in that case, the influence of the narcotic in the part was propagated through the nerves; but the experiments of Fontana and Wernschedt were sufficient to prove the incorrectness of that opinion. In the experiments of the latter, when the nervus vagus was divided on both sides, opium, introduced into the stomach, produced its influence upon the habit as readily as when these nerves were entire; and this result was confirmed by the experiments of Brodie, Emmert, Delille, Wedemeyer, and Viridet.

These facts were sufficient to demonstrate that narcotics endermically administered acted both locally and generally; and from experiments which he, Dr. T., had made, he had no hesitation in affirming, that when salts of morphia were applied to a blistered, or otherwise denuded surface, the narcotic might either exhaust itself locally, or it might be taken into the circulation, and operate upon the cerebro-spinal centres as rapidly as when it was introduced into the stomach. The time required for the imbibition of a narcotic thus applied, in order to reach the capillaries, and to enter the circulation, was stated by Müller, in his "Elements of Physiology," to be less than a second; and as the blood circulated through the whole body in half a minute, if Herring's calculation was correct; or from one to two minutes, according to others, it was not assuming too much to suppose that a narcotic placed in contact with a part freed from epidermis, might be distributed through the circulating system in a space of time between half a minute and two minutes. In some of the instances in which finely powdered hydrochlorate of morphia was sprinkled over a blistered surface, the influence of the narcotic was felt in the head in less than a minute. He might here enumerate the symptoms which indicated the general influence of the narcotic, thus applied, upon the cerebro-spinal centres; but he preferred leaving these to the details of the cases, illustrative of the practice, which he was selected to lay before the students. He might however, mention, that when the nerves were topically affected, as in neuralgia, the general symptoms scarcely ever displayed themselves.

Such was the physiological action of narcotics applied to the denuded surface of the body, which had hitherto been recorded; but there was another action produced by at least one of these, namely, hydrochlorate of morphia, which had not, to his knowledge, been previously observed, and to which much of the advantages derived from its application, in a therapeutical point of view, was to be attributed. He referred to the production of a papular eruption, terminating in pustules, which spread from the immediate vicinity of the part in which the hydrochlorate was applied, all over the body; and it was more or less attended with œdema. In some instances the swelling when the blistered surface was in the vicinity of the head, had been so considerable as to close the eyes, in a manner similar to that which occurs in erysipelas of the head. It had also been attended with some degree of fever; and in a few instances delirium had displayed itself when the eruption had reached its acme. It seemed to operate as a most powerful and efficient counter-irritant, without, apparently, interfering with the narcotic influence of the hydrocholorate. The eruption was a pustular one, and he had observed that the relief was most obvious as soon as the pustules were fairly formed; indeed, so striking had been the beneficial influence of this eruption, that he was inclined to accord with the remark of Dr. Jenner, that "every pimple with a vesiculated head had an errand to perform for the benefit of the constitution."

He had selected the preceding cases from a great number. The first three were intended to illustrate the local influence of the endermic application of the salts of morphia; the other two, their general influence. His object in bringing forward these cases was, to direct attention to the endermic practice which promised many advantages; and which only required to be investigated to gain the support of British practitioners.

*Lancet.*

---

*Sentence of Chauncey, the Botanical Physician.*—Chauncey, the Botanical Physician, found guilty of the murder of Eliza Sowers of Manayunk, in procuring an abortion, and whose trial was reported at length in No. 5, of the Examiner, has been sentenced to five years imprisonment. A motion for a new trial was overruled.

